# Supplementary material for: A Cross-Species Analysis Reveals a General Role for Piezo2 in Mechanosensory Specialization of Trigeminal Ganglia from Tactile Specialist Birds
Source: Cell Rep. Author manuscript; Available in PMC 2019 Mar 16. (PMC6420409; doi:10.1016/j.celrep.2019.01.100)
Supplement: 1 [file NIHMS1522168-supplement-1.pdf]

Cell Reports, Volume *26*

Supplemental Information

A Cross-Species Analysis Reveals a General Role for Piezo2 in Mechanosensory Specialization of Trigeminal Ganglia from Tactile Specialist Birds

Eve R. Schneider, Evan O. Anderson, Viktor V. Feketa, Marco Mastrotto, Yury A. Nikolaev, Elena O. Gracheva, and Sviatoslav N. Bagriantsev

Supplemental Data

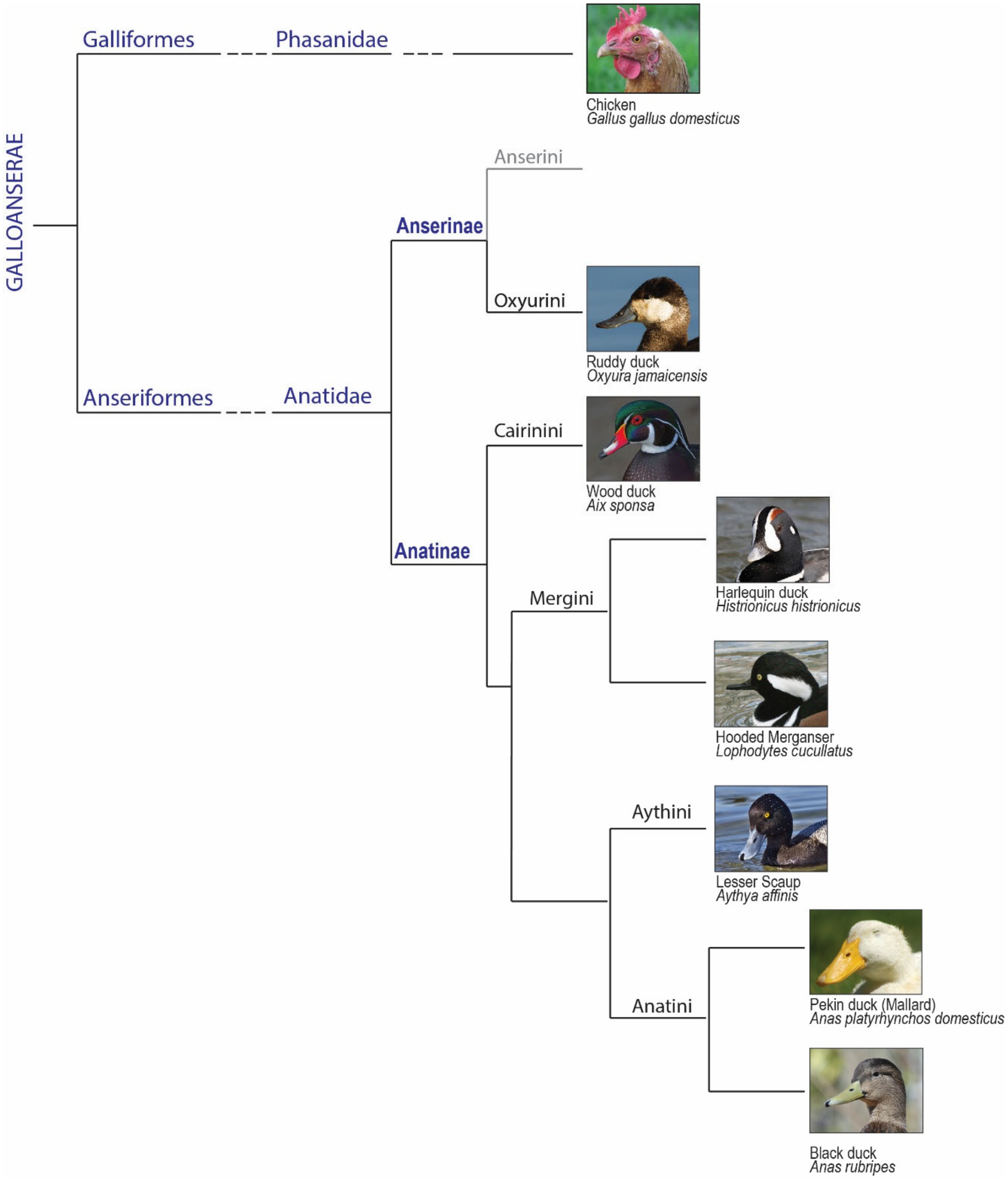

Figure S1. Phylogeny of bird species used in this study. Related to Figure 1. A simplified phylogenetic tree showing evolutionary relationship between the birds used in this study, without time representation. Anatids are representative of 2 subfamilies, 5 tribes and 6 genera. Tribes for which there are single species are not shown. The tree is adapted from (Buckner et al., 2018; Gonzalez et al., 2009). Photos courtesy of: Judy Gallagher (Wood, image cropped, CC BY 2.0); Frank Schulenburg (Ruddy, image cropped, CC BY-SA 3.0); Peter Massas (Harlequin, image cropped. CC BY-SA 2.0); Dick Daniels (Hooded Merganser and Black, image cropped, CC-BY-SA-3.0); Alan D. Wilson (Lesser Scaup, image cropped, CC-BY-SA-2.5); Eve Schneider (Pekin), Bagriantsev lab; Filip Maljković (Chicken, public domain).

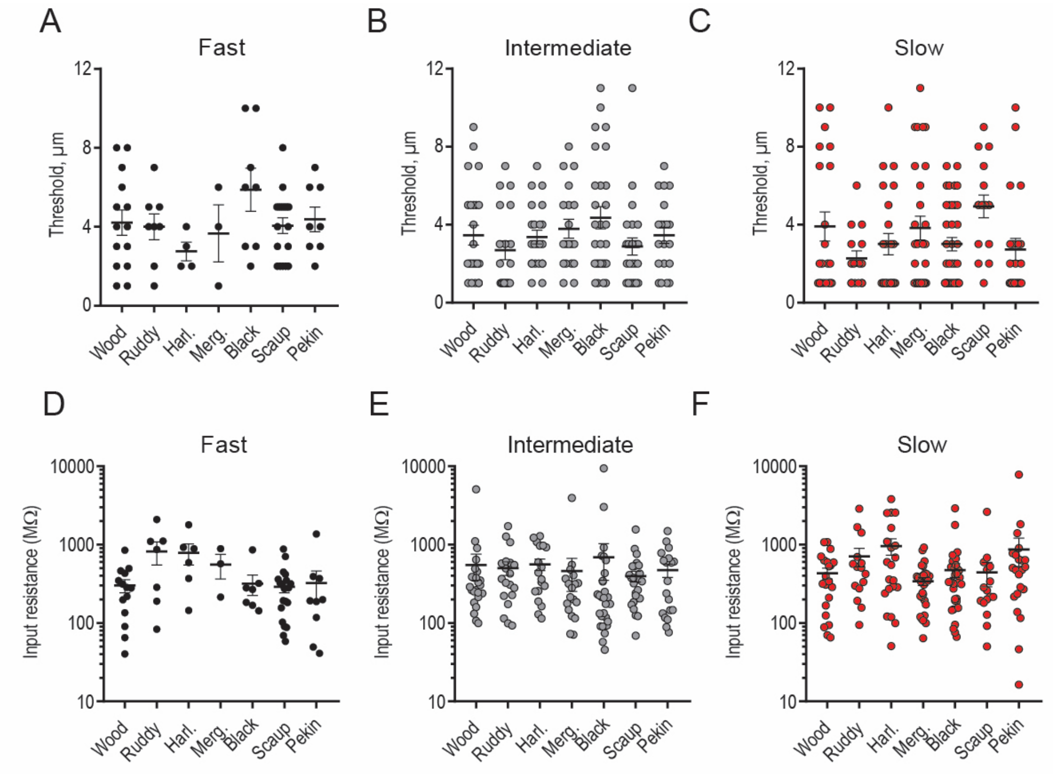

Figure S2. Threshold of mechano-current activation and input resistance of duck TG neurons. Related to Figure 1. (A–C) Quantification of the apparent MA current activation threshold from dissociated duck TG neurons. Whole-cell MA currents were elicited in response to mechanical indentation with a glass probe at E_{hold} = -74.6 mV. The threshold was determined as the first indentation to elicit a peak current greater than background noise, typically at least 50 pA above averaged baseline. Data shown as mean ± SEM. Statistical analysis (one-way ANOVA): P=0.317 (A), P=0.205 (B), P=0.063 (C). Input resistance of trigeminal neurons with the indicated types of MA current. Data shown as mean ± SEM, collected from 2-6 birds for each species. Statistical analysis (Kruskal-Wallis test): P=0.102 (D), P=0.150 (E), P=0.203 (F).

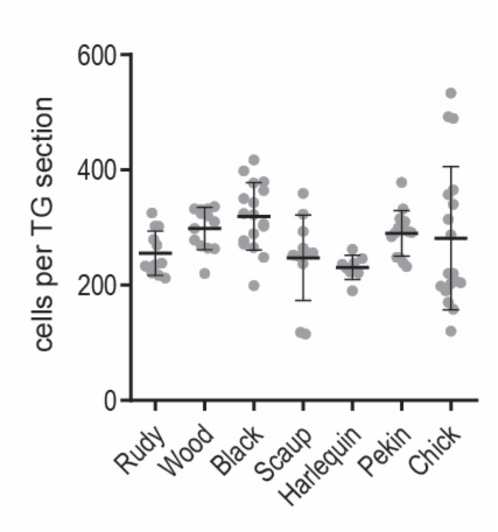

Figure S3. **Trigeminal ganglia from ducks and chicken have a similar total number of neurons. Related to Figure 2.** Quantification of the total number of cells per random TG section from indicated species. Data shown as mean ± SD, collected from ≥2 birds for each species. P≥0.05 for all sample pairs (Tukey multiple comparisons test).

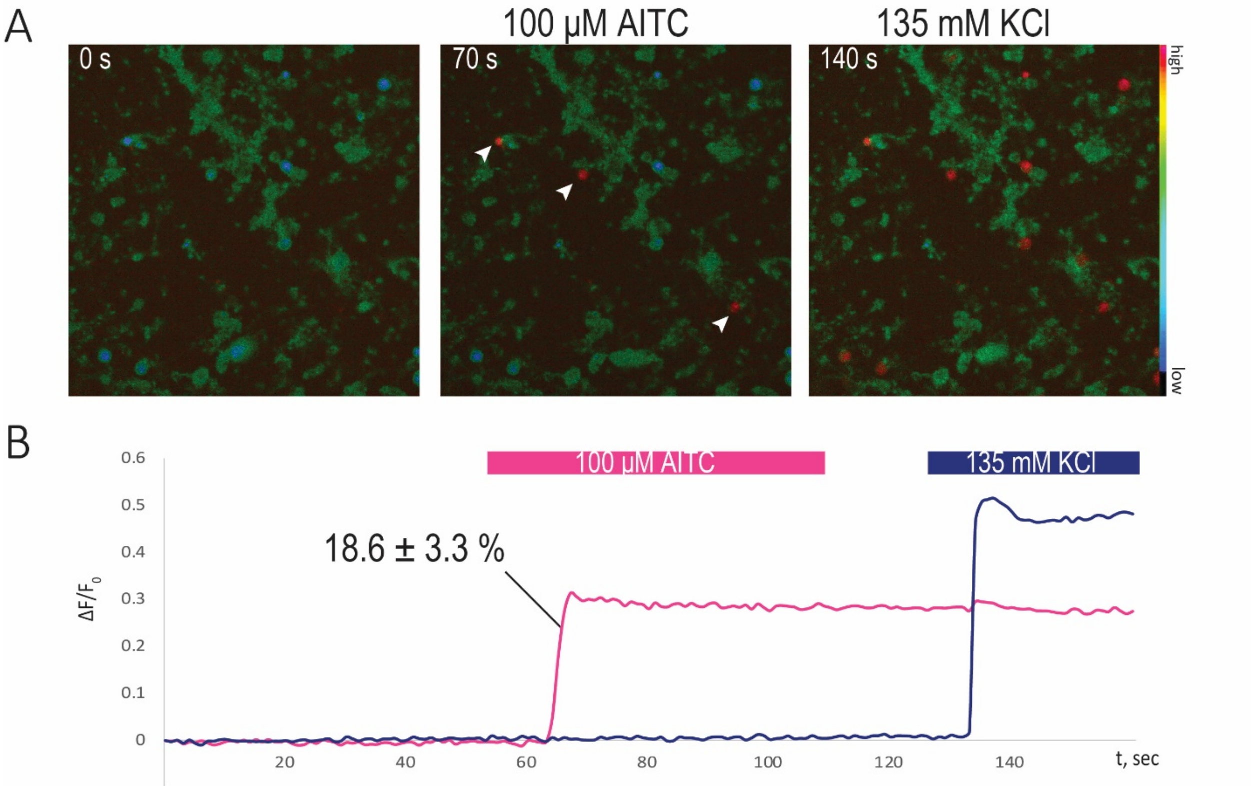

Figure S4. **Quantification of the abundance of polymodal nociceptors in Pekin duck TG. Related to Figure 4.** (A) Representative fields of view of Fura-2AM live-cell ratiometric calcium imaging of dissociated duck TG neurons in response to the application of 100 µM AITC. Arrowheads denote AITC-sensitive cells. 135 mM KCl is used to visualize all neurons in the field of view. Color coding denotes lowest and highest ratios from bottom to top. (B) Exemplar traces of calcium responses in an AITC-sensitive and insensitive cell, from the images of duck TG neurons above (mean ± SEM, n = 158 neurons from 5 coverslips, from 4 animals).
