# Supplementary material for: A Cross-Species Analysis Reveals a General Role for Piezo2 in Mechanosensory Specialization of Trigeminal Ganglia from Tactile Specialist Birds
Source: Cell Rep. Author manuscript; Available in PMC 2019 Mar 16. (PMC6420409; doi:10.1016/j.celrep.2019.01.100)
Supplement: 3 [file NIHMS1522168-supplement-3.pdf]

Cell Reports

Report

A Cross-Species Analysis Reveals a General Role for Piezo2 in Mechanosensory Specialization of Trigeminal Ganglia from Tactile Specialist Birds

Graphical Abstract

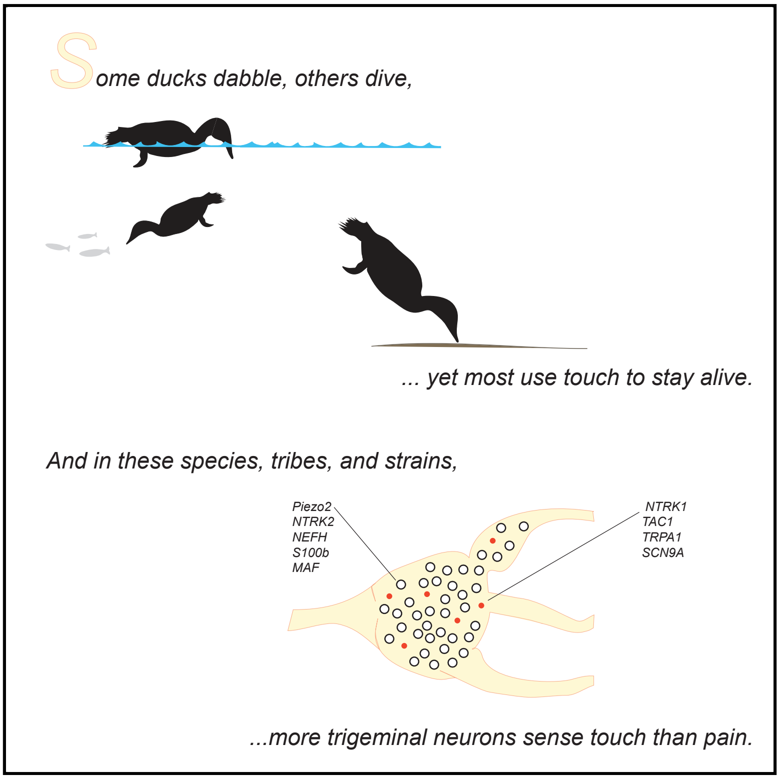

Highlights

- A cross-species analysis reveals common principles of mechanosensory specialization
- Mechanoreceptor expansion occurs via increase in neurons with slow mechanocurrent
- A trade-off between mechanoreceptors and other sensory neurons is a general trend
- Piezo2 plays a general role in mechanosensory specialization of trigeminal ganglia

Authors

Eve R. Schneider, Evan O. Anderson, Viktor V. Feketa, Marco Mastrotto, Yury A. Nikolaev, Elena O. Gracheva, Sviatoslav N. Bagriantsev

Correspondence

elena.gracheva@yale.edu (E.O.G.),
slav.bagriantsev@yale.edu (S.N.B.)

In Brief

Schneider et al. perform a cross-species analysis of somatosensory neurons from tactile specialist birds. The study reveals a trade-off in the expansion of Piezo2-containing neuronal touch receptors at the expense of temperature and pain receptors as part of a general mechanism that accompanies mechanosensory specialization.

Schneider et al., 2019, Cell Reports *26*, 1979–1987
February 19, 2019 © 2019 The Author(s).
https://doi.org/10.1016/j.celrep.2019.01.100

CellPress

Cell Reports
Report

OPEN ACCESS
CellPress

A Cross-Species Analysis Reveals a General Role for Piezo2 in Mechanosensory Specialization of Trigeminal Ganglia from Tactile Specialist Birds

Eve R. Schneider,[1,4] Evan O. Anderson,[1] Viktor V. Feketa,[1,2,3] Marco Mastrotto,[1,2,3] Yury A. Nikolaev,[1] Elena O. Gracheva,[1,2,3,*] and Sviatoslav N. Bagriantsev[1,5,*]
[1]Department of Cellular and Molecular Physiology, Yale University School of Medicine, 333 Cedar St., New Haven, CT 06510, USA
[2]Department of Neuroscience, Yale University School of Medicine, 333 Cedar St., New Haven, CT 06510, USA
[3]Program in Cellular Neuroscience, Neurodegeneration and Repair, Yale University School of Medicine, 333 Cedar St., New Haven, CT 06510, USA
[4]Present address: Department of Biology, University of Kentucky, Lexington, KY 40506, USA
[5]Lead Contact
*Correspondence: elena.gracheva@yale.edu (E.O.G.), slav.bagriantsev@yale.edu (S.N.B.)
https://doi.org/10.1016/j.celrep.2019.01.100

SUMMARY

A major challenge in biology is to link cellular and molecular variations with behavioral phenotypes. Here, we studied somatosensory neurons from a panel of bird species from the family Anatidae, known for their tactile-based foraging behavior. We found that tactile specialists exhibit a proportional expansion of neuronal mechanoreceptors in trigeminal ganglia. The expansion of mechanoreceptors occurs via neurons with intermediately and slowly inactivating mechanocurrent. Such neurons contain the mechanically gated Piezo2 ion channel whose expression positively correlates with the expression of factors responsible for the development and function of mechanoreceptors. Conversely, Piezo2 expression negatively correlates with expression of molecules mediating the detection of temperature and pain, suggesting that the expansion of Piezo2-containing mechanoreceptors with prolonged mechanocurrent occurs at the expense of thermoreceptors and nociceptors. Our study suggests that the trade-off between neuronal subtypes is a general mechanism of tactile specialization at the level of somatosensory system.

INTRODUCTION

Mechanosensory neurons from trigeminal ganglia (TG) mediate the initial detection of the mechanical stimuli in the bill, tongue, and oral cavity and are essential for tactile-based foraging. Ducks employ various foraging strategies, including dabbling, straining, filtering, pecking, and grazing (Avilova, 2018; Avilova et al., 2018; Berkhoudt, 1980; McNeil et al., 1992; Saxod, 1978; Zweers, 1977). Wood ducks (*Aix sponsa*) often feed by visually guided pecking, searching for food items such as acorns and seeds in shallow wetlands (Drobney and Fredrickson, 1979). Ruddy ducks (*Oxyura jamaicensis*) are divers, feeding by straining benthic material underwater (Tome and Wrubleski, 1988). Harlequin ducks (*Histrionicus histrionicus*) and hooded mergansers (*Lophodytes cucullatus*) obtain most of their food by diving, often under conditions of poor visibility. Lesser scaups (*Aythya affinis*) are diver-pursuers, but also rely on the tactile location of food (Tome and Wrubleski, 1988). The Pekin duck (*Anas platyrhynchos domesticus*), a domesticated descendant of the mallard, and its close relative the black duck (*Anas rubripes*) are probably the most sophisticated tactile foragers and are the most well studied (Zweers, 1977). While it is difficult to compare physiological sensitivities to touch among the duck species directly, Pekin and black ducks are tactilely guided dabblers known to possess an exceptional ability to forage almost entirely based on the sense of touch. In controlled experiments, Pekin ducks were able to catch fast-moving tadpoles in complete darkness. The application of anesthetic on the bill surface suppresses foraging efficiency, consistent with a tactile-based mechanism (Avilova, 2017). Some species are nocturnal foragers (black, mallard, ruddy, and scaup), while others are primarily diurnal (harlequin and merganser) or crepuscular (wood) (McNeil et al., 1992).

Food preferences and foraging behaviors of these species suggest that some are more capable tactile foragers than others, which could be reflected in the composition and functional properties of somatosensory neurons in TG. We tested this by performing a correlative analysis of the abundance of mechanosensory neuronal types in TG, the proportion of neurons expressing the mechanogated ion channel Piezo2, and the expression levels of markers of mechanoreceptors versus thermo- and nociceptors in TG from seven species of Anatidae from six genera (Figures 1A and S1). Because functional specialization of sensory neurons in ducks completes before hatching, we used tissues isolated from late-stage embryos (Saxod, 1978; Schneider et al., 2017).

RESULTS

Mechanoreceptor Expansion in Duck TG Occurs via an Increase in Neurons with Intermediate and Slow Mechanocurrent

To quantify the proportion of mechanosensitive neurons, we used whole-cell electrophysiology to record mechanically

Check for updates

This is an open access article under the CC BY license (http://creativecommons.org/licenses/by/4.0/).

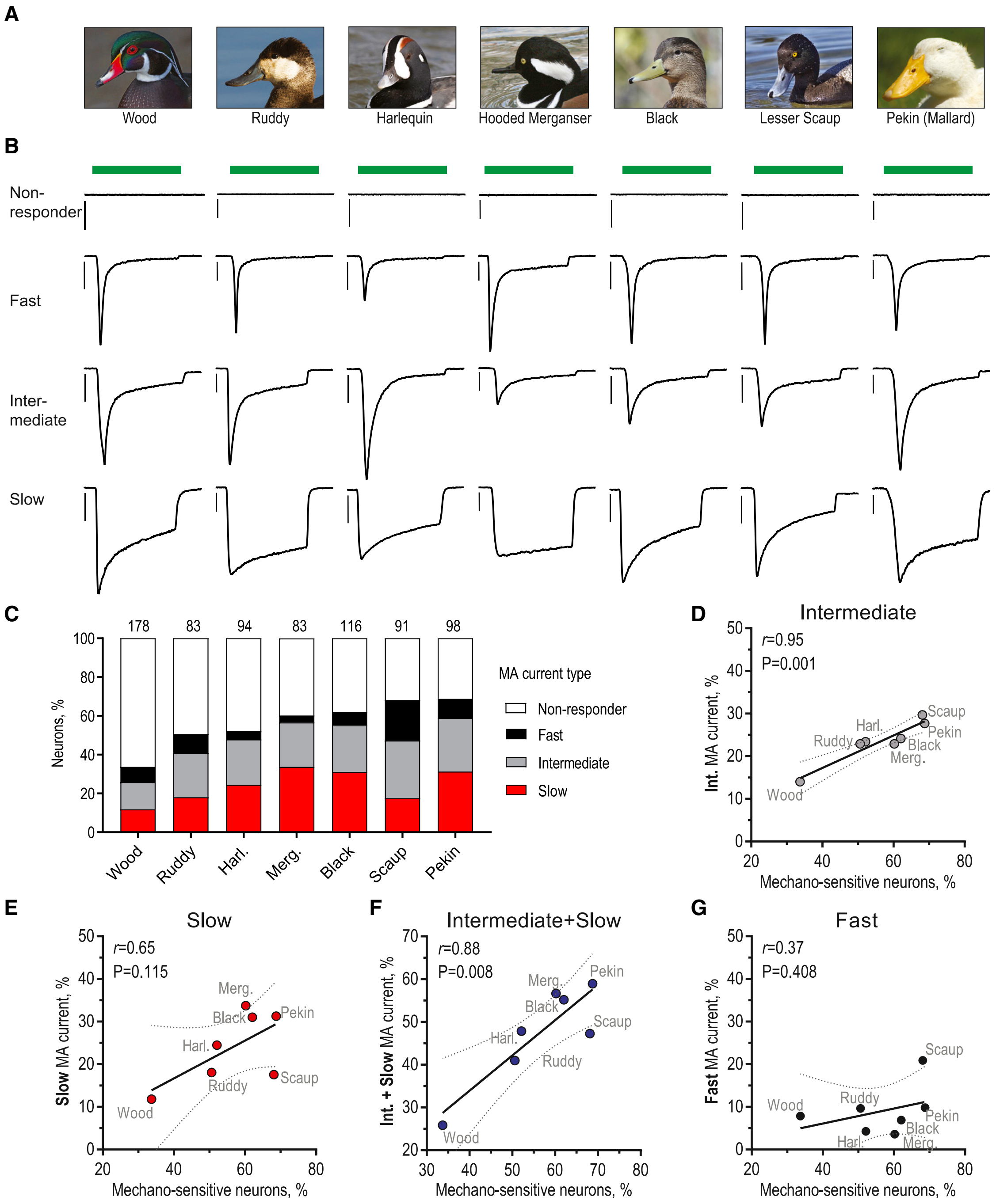

Figure 1. Expansion of Trigeminal Mechanoreceptors with Slow and Intermediate Mechanocurrent

(A) Images of duck species used in the study. Photos courtesy of Judy Gallagher (wood, image cropped, CC BY 2.0), Frank Schulenburg (ruddy, image cropped, CC BY-SA 3.0), Peter Massas (harlequin, image cropped, CC BY-SA 2.0), Dick Daniels (hooded merganser and black, image cropped, CC BY-SA-3.0), Alan D. Wilson (lesser scaup, image cropped, CC BY-SA-2.5), and Eve Schneider (Pekin), Bagriantsev lab.

(B) Exemplar whole-cell MA current traces recorded in dissociated duck TG neurons in response to a 150 ms mechanical indentation (green bar) with a glass probe for a depth of 3–15 μm at $E_{hold} = -74.6$ mV. Scale bar, 1 nA.

(C) Quantification of the proportions of neurons with the fast, intermediate, and slow MA current types (χ^2 test; $p < 0.0001$). Numbers indicate total numbers of neurons analyzed for each species.

(D–G) Correlation between the percentage of mechanosensitive neurons and the percentage of neurons with intermediate (D), slow (E), intermediate and slow (F), and fast (G) MA current, fitted to the linear equation. *r* is the Pearson correlation coefficient, P is the probability that observed variation results from random sampling, and dotted lines show the 95% confidence interval. Data were collected from 2–6 birds for each species.

See also Figures S1 and S2.

activated (MA) current from dissociated TG in response to stimulation with a glass probe (McCarter et al., 1999). We found that the abundance of neurons responding to mechanical stimulation varied significantly across duck species, from lowest in wood duck to highest in Pekin duck (33.7% and 68.8% of all TG neurons, respectively; χ^2 test; $p < 0.0001$) (Figures 1B and 1C). However, even in wood duck, the proportion of mechanoreceptors was higher than that found earlier in chicken (19.8% of all TG neurons), a strictly visually foraging bird (Schneider et al., 2017). These data show that the proportional expansion of mechanosensitive neurons in TG is a general phenomenon among Anatidae waterfowl, consistent with the idea that many duck species are tactilely guided foragers.

The duration of MA current determines the amount of depolarizing ionic flux, serving as a critical determinant of neuronal mechanosensitivity. In vertebrates, somatosensory neurons exhibit one of three types of MA current: with fast, intermediate, or slow kinetics of inactivation (inactivation constant: $\tau_{inact} <$ 10 ms for fast, τ_{inact} = 10–30 ms for intermediate, $\tau_{inact} >$ 30 ms for slow) (Coste et al., 2007, 2010; Hu and Lewin, 2006; Rugiero et al., 2010; Schneider et al., 2014, 2017; Wetzel et al., 2007). The three types of MA current are mediated by more than one mechanically gated ion channel (Ranade et al., 2015). We aimed to determine which neuronal population, as defined by its characteristic type of MA current, contributed most to the increase in the proportion of trigeminal mechanoreceptors among the duck species. We found a strong positive linear correlation between total fraction of mechanosensitive neurons and neurons with intermediate and slow MA current (total versus intermediate, Pearson r = 0.95, p = 0.001; total versus slow, r = 0.65, p = 0.115; total versus intermediate + slow, r = 0.88, p = 0.008) (Figures 1D–1F). The proportion of neurons with fast MA current, which mediates the detection of light touch in mice, did not correlate with mechanoreceptor expansion (total versus fast, r = 0.37, p = 0.408) (Figure 1G) (Ranade et al., 2014). The number of active channels on the surface and their sensitivity to stimulation affect the apparent mechanocurrent activation threshold, defined as the minimal indentation that elicits MA current. We found that the threshold remained unchanged in all groups, suggesting that the expansion of neurons with intermediate and slow MA current is not accompanied by a significant change in sensitivity or an increase in expression of the underlying ion channels (Figures S2A–S2C). We also did not detect a difference in input resistance among comparable groups of neurons from the seven duck species (Figures S2D–S2F).

Our data suggest that the increase in the proportion of trigeminal mechanoreceptors across the seven duck species occurs via an expansion of neurons with intermediate and slow MA current. Slowly and intermediately inactivating mechanosensitive currents provide longer-lasting depolarization than a fast inactivating current of comparable amplitude and may increase the chance of action potential firing in response to mechanical stimulation. Thus, the high proportion of neurons with slow and intermediate mechanosensitive currents in TG is expected to potentiate mechanical sensitivity at the level of individual sensory neurons.

Neurons with Intermediate and Slow Mechanocurrent Positively Correlate with Abundance of Piezo2$^+$ Cells

The mechanically gated ion channel Piezo2 is the only known mechanotransducer in vertebrate somatosensory neurons responsible for the detection of touch (Anderson et al., 2017; Ranade et al., 2015). In mice, the deletion of Piezo2 selectively obliterates fast MA current (Ranade et al., 2014), whereas in Pekin duck, the depletion of Piezo2 leads to downregulation of intermediate and slow MA current (Schneider et al., 2017). This suggests that the contribution of Piezo2 to neuronal mechanosensitivity varies by species and that the kinetics of Piezo2 inactivation could be part of the mechanism supporting mechanosensory potentiation in tactile foraging animals. To test this, we performed a correlative analysis of the proportion of mechanosensitive trigeminal neurons and neurons that express Piezo2, as determined by RNA *in situ* hybridization, in TG of six duck species (Figure 2). We found a strong positive correlation between the percentage of Piezo2$^+$ neurons and the percentage of neurons with intermediate and slow MA current (Piezo2 versus intermediate + slow, Pearson r = 0.83, p = 0.040; Piezo2 versus intermediate, r = 0.73, p = 0.101; Piezo2 versus slow, r = 0.74, p = 0.095) (Figures 3A–3C). Neurons with fast MA current, however, showed no correlation with Piezo2$^+$ cells (Piezo2 versus fast, r = 0.19, p = 0.717) (Figure 3D). The total number of neurons per TG section did not differ among the species (Figure S3). Altogether, our data suggest a general mechanism of mechanoreceptor expansion in TG of tactile foraging ducks via an increase in the proportion of Piezo2$^+$ neurons with intermediate and slow MA current. However, it is possible that neurons without Piezo2 or neurons expressing another unknown mechanosensitive ion channel together with Piezo2 also contribute to mechanoreceptor expansion.

PIEZO2 Expression Negatively Correlates with the Expression of Nociceptive Markers

Previously, we determined that the abundance of Piezo2-expressing mechanoreceptors is higher in Pekin duck TG than in chicken (*Gallus domesticus*), suggesting that mechanoreceptor expansion in tactile foragers could occur at the expense of other functional neuronal groups, such as nociceptors (Schneider et al., 2017). To functionally validate the observed decrease in nociceptors, we performed live-cell ratiometric calcium imaging of Pekin duck TG neurons treated with allyl isothiocyanate (AITC), a specific agonist of TRPA1, an ion channel specific to polymodal nociceptors in birds (Saito et al., 2014). We found that 18.6% ± 3.3% (mean ± SEM, n = 158 cells) of neurons responded to AITC (Figure S4), a markedly lower population than the 34% of TRPA1-positive neurons in chicken (Saito et al., 2014). Given functional validation of previous *in situ* hybridization data, we sought to understand whether the trade-off between mechanoreceptors and other neuronal types, mainly thermo- and nociceptors, is a general strategy employed by tactile foraging birds. To do this, we performed transcriptome analysis of trigeminal ganglia isolated from six duck species and domestic chicken and determined a correlation between the expression of *PIEZO2* and that of well-established markers of mechanoreceptors and nociceptors.

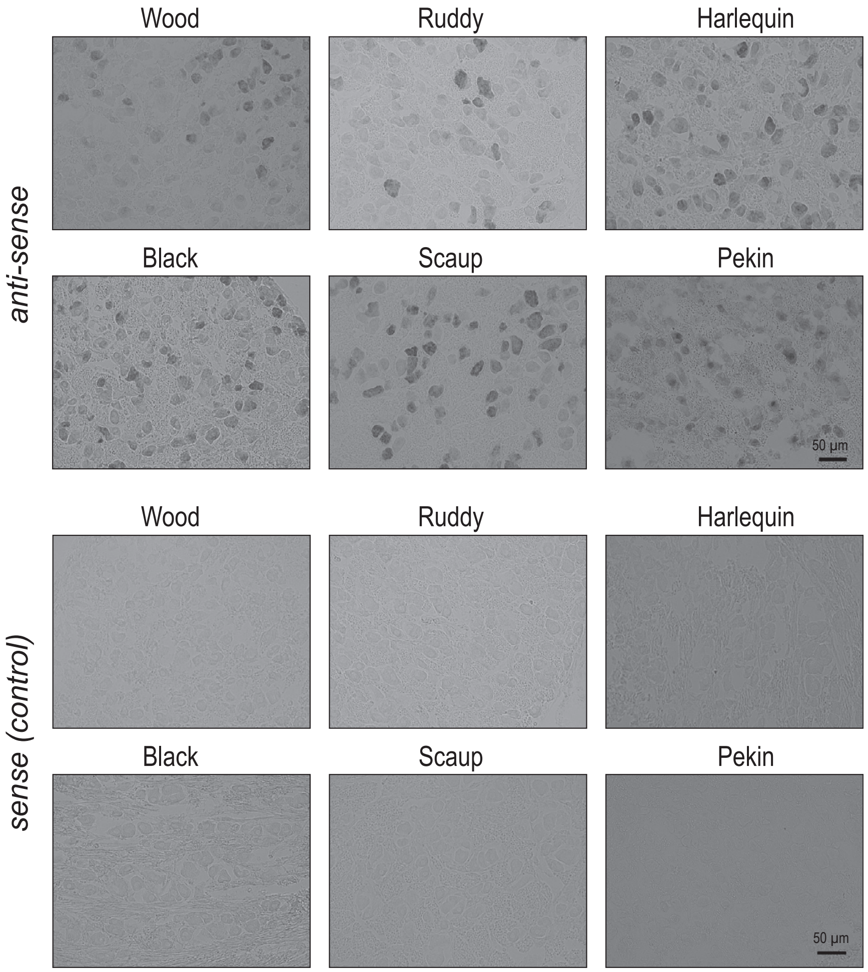

Figure 2. Piezo2 Expression in Duck TG
Representative images of RNA *in situ* hybridization in TG of indicated bird species with anti-Piezo2 (anti-sense) and control (sense) probes. Data were collected from 2–6 birds for each species.

We found a strong positive correlation between the expression of *PIEZO2* and *NTRK2* (TrkB), a receptor tyrosine kinase, and *MAF* (c-MAF), a transcription factor, both responsible for proper development of mechanoreceptors (*PIEZO2* versus *NTRK2*, Pearson $r = 0.77$, $p = 0.042$; *PIEZO2* versus *MAF*, $r = 0.77$, $p = 0.042$) (Figure 4A; Table S1) (Dhandapani et al., 2018; Kobayashi et al., 2005; Lallemend and Ernfors, 2012; Wende et al., 2012). Similarly, *PIEZO2* positively correlated with the mechanoreceptor marker heavy-chain neurofilament *NEFH* (NF200) and the calcium-binding protein S100β (*PIEZO2* versus *NEFH*, $r = 0.72$, $p = 0.069$; *PIEZO2* versus *S100β*, $r = 0.86$, $p = 0.013$) (Figure 4A; Table S1). In duck bill skin, touch is detected by Grandry and Herbst corpuscles, the analogs of the mammalian Meissner and Pacinian corpuscles, respectively. The corpuscles are tuned to detect transient touch and vibration and are innervated by rapidly adapting Aβ-type trigeminal mechanoreceptors (Gottschaldt, 1974; Schneider et al., 2017). *NTRK2* (TrkB) is critical for rapidly adapting mechanoreceptor development and function and is expressed in nerve terminals and lamellar cells of Pacinian and Meissner corpuscles (Cabo et al., 2015; Calavia et al., 2010; Dhandapani et al., 2018). In mice, the deletion of *MAF* (c-MAF) decreases the number of Meissner and Pacinian corpuscles and attenuates corpuscle function (Wende et al., 2012). In humans and mice, *S100β* is expressed in both neuronal and somatic components of Meissner and Pacinian corpuscles (Fleming et al., 2016; García-Suárez et al., 2009; Gonzalez-Martinez et al., 2003; Heidenreich et al., 2011; Luo et al., 2009). Thus, the positive correlation between *PIEZO2* with these molecules is consistent with their role in light touch detection by rapidly adapting mechanoreceptors.

Conversely, *PIEZO2* expression strongly and negatively correlated with *NTRK1* (TrkA), a receptor tyrosine kinase required for proper development of most C-type nociceptors and temperature receptors (*PIEZO2* versus *NTRK1*, $r = -0.79$, $p = 0.034$) (Figure 4B; Table S1) (Lallemend and Ernfors, 2012). We also found a strong negative correlation between *PIEZO2* and *TAC1*, the precursor of the nociceptive neuropeptide substance P, and *TRPA1*, the principal sensor of heat in birds and reptiles (*PIEZO2* versus *TAC1*, $r = -0.81$, $p = 0.027$; *PIEZO2* versus *TRPA1*, $r = -0.82$, $p = 0.023$) (Gracheva and Bagriantsev, 2015; Kurganov et al., 2014; Saito et al., 2014). Furthermore, *PIEZO2* expression negatively correlated with the voltage-gated sodium channel *SCN9A* ($Na_v1.7$), a major contributor to action potential generation in nociceptors (*PIEZO2* versus *SCN9A*, $r = -0.87$, $p = 0.010$) (Figure 4B; Table S1) (Minett et al., 2012; Tanaka et al., 2017; Yang et al., 2017). Altogether, these data strongly support the notion that trigeminal mechanoreceptor expansion occurring at the expense of nociceptors and thermoreceptors is a general strategy employed by tactile foraging species.

DISCUSSION

In this study, we combined electrophysiology, histochemistry, and transcriptomics to analyze trigeminal ganglia from a panel of tactile foraging birds to identify cellular and molecular prerequisites of mechanosensory specialization. Our study reveals several key trends: (1) the proportion of mechanosensitive neurons in TG is higher in tactile specialist ducks than in visually foraging birds such as chicken (Schneider et al., 2014, 2017), (2) the proportional expansion of trigeminal mechanoreceptors occurs via neurons that exhibit MA current with intermediate

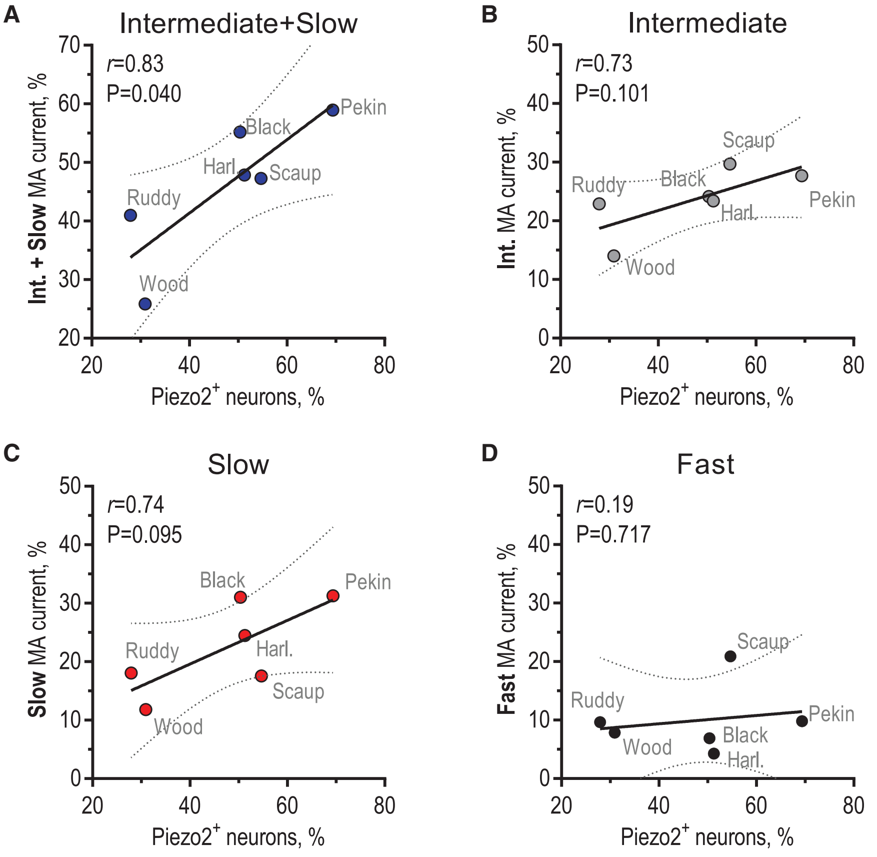

Figure 3. Neurons with Intermediate and Slow Mechanocurrent Positively Correlate with Abundance of Piezo2$^+$ Cells

(A–D) Correlation between the percentage of Piezo2-expressing neurons in duck TG (shown as the average from 1,610–3,876 total neurons from 7–17 TG sections) and the percentage of neurons with intermediate and slow (A), intermediate (B), slow (C), and fast (D) MA current, fitted to the linear equation. *r* is the Pearson correlation coefficient, P is the probability that observed variation results from random sampling, and dotted lines show the 95% confidence interval. Data were collected from 2–6 birds for each species.

See also Figure S3.

and slow kinetics of inactivation and express the Piezo2 ion channel, and (3) Piezo2 expression positively correlates with markers of mechanosensitivity and negatively correlates with markers of thermo- and nociception. These trends suggest a common mechanism employed by Anatidae waterfowl to potentiate mechanosensation in the bill.

Tactile-based feeding behavior implies that an organism can preferentially rely on using the sense of mechanical touch for foraging rather than other senses, such as olfaction and vision. Pekin duck is particularly adept at this task, because it is able to forage in complete darkness, solely relying on mechanosensitivity. As such, and for logistical reasons, Pekin ducks present an attractive animal model with which to study the cellular and molecular basis of the sense of touch in glabrous skin (Schneider et al., 2014, 2017). Here, we show that Pekin duck has the highest proportion of mechanically sensitive neurons, Piezo2-expressing neurons, and the highest level of *PIEZO2* mRNA in TG among the seven duck species tested. Our findings agree with the earlier observations that duck bill skin contains a high density (up to 170 per square millimeter) of Grandry (Meissner) and Herbst (Pacinian) mechanosensory corpuscles (Berkhoudt, 1980; Schneider et al., 2017), which require rapidly adapting trigeminal mechanoreceptors for development and function (Gottschaldt, 1974; Saxod, 1996).

Unlike Pekin duck, wood duck often uses the pecking technique for foraging, which primarily relies on visual cues. Accordingly, in contrast to the wide bill of Pekin duck, wood duck has a narrow, beak-like bill, most suitable for grabbing small objects such as acorns, their preferred food. The smaller bill also implies a smaller tactile area. Consistently, we found that wood duck has the lowest proportion of mechanoreceptors and Piezo2-expressing neurons in TG. We therefore speculate that in this sense, wood duck is closer to visual foragers such as chicken than to tactile foragers such as Pekin and black ducks. However, all ducks employ tactile-based foraging to some extent and exhibit more abundant representation of mechanoreceptors than the strictly visually foraging chicken.

Our results also indicate the existence of a trade-off in the increasing proportions of Piezo2$^+$ mechanoreceptors that comes at the expense of other groups of sensory neurons. While the exact mechanism is unclear, it involves a differential expression of neurotrophic growth factor receptors *NTRK2* (TrkB) and *NTRK1* (TrkA), which drive the differentiation of neuronal precursors into mechanoreceptors versus nociceptors and thermoreceptors, respectively (Lallemend and Ernfors, 2012). In both late-embryonic and adult Pekin duck TG, *NTRK2*$^+$ neurons greatly outnumber *NTRK1*$^+$ cells (Schneider et al., 2017). Here, our correlative analysis from seven bird species shows a significant positive correlation of the expression of *PIEZO2* with *NTRK1* and a negative correlation with *NTRK2*, suggesting that the trade-off mechanism is a general phenomenon among Anatidae. Although most Piezo2$^+$ neurons in duck TG are mechanoreceptors, a small fraction could represent nociceptors, in agreement with the findings that in addition to its major role in light touch detection, Piezo2 contributes to the development of mechanical allodynia and hyperalgesia (Murthy et al., 2018; Prato et al., 2017; Szczot et al., 2017, 2018). Whether the proportional reduction in nociceptors in duck TG correlates with physiological sensitivity to these stimuli is unknown and remains to be determined. It is possible to envision that even a small number of receptors could be sufficient to detect minute changes in temperature or to signal pain.

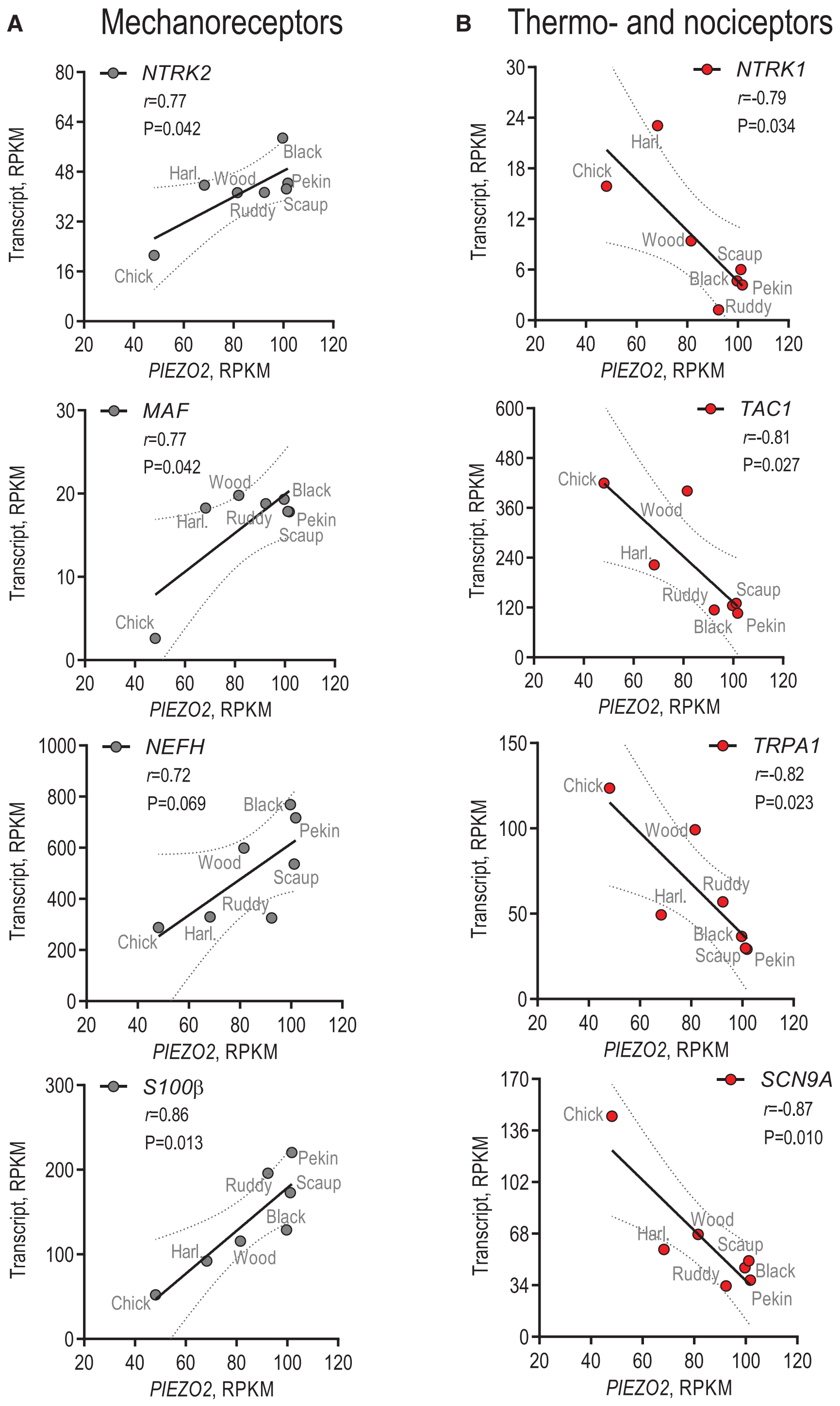

Figure 4. Correlation of *PIEZO2* Expression with Markers of Mechanoreceptors, Thermoreceptors, and Nociceptors

(A and B) Shown are correlations between the levels of expression of *PIEZO2* and markers of mechanoreceptors (A) or thermoreceptors and nociceptors (B), fitted to the linear equation. Data shown as the average from TGs from three birds for each species. RPKM, reads per kilobase of transcript length normalized per million of total reads. *r* is the Pearson correlation coefficient, P is the probability that observed variation results from random sampling, and dotted lines show the 95% confidence interval.

See also Figure S4 and Table S1.

With their high density of corpuscles in the bill and sophisticated feeding behavior (Zweers, 1977), many Anatidae birds are among the most capable tactile specialists (Schneider et al., 2016). In this sense, ducks rival the undisputed champion in tactile foraging, the star-nosed mole (*Condylura cristata*). The mole has 22 sensory appendages surrounding the nostrils covered with glabrous skin and containing hundreds of mechanosensory end organs per square millimeter (Catania, 2011; Catania and Remple, 2005). Behavioral studies showed that capsaicin, a chemical that activates mammalian nociceptors, fails to elicit nocifensive response when applied to the star organ, but not to the hindpaw. Functional and histological analysis of trigeminal ganglia versus dorsal root ganglia agree with behavioral data, suggesting the possibility of a TG-specific expansion of mechanoreceptors at the expense of thermo- and nociceptors (Gerhold et al., 2013). Thus, the trends we identified here at the level of primary afferents in Anatidae could be true for tactile specialists from other clades of vertebrates, providing a fascinating example of convergent evolution (Schneider et al., 2016).

The magnitude and duration of MA current are important determinants of mechanically evoked excitability. In mouse somatosensory neurons, Piezo2 mediates MA current with fast kinetics of inactivation (Anderson et al., 2017; Ranade et al., 2014, 2015). In Pekin TG, the down-regulation of Piezo2 diminishes the amplitude of intermediately and slowly inactivating MA current, suggesting that the channel has evolved to produce a higher degree of depolarization in response to a mechanical stimulus of the same magnitude (Schneider et al., 2017). Here, our analysis of seven duck species reveals a significant positive correlation between the abundance of Piezo2-expressing neurons and the number of neurons with intermediate and slow MA current. These data suggest the existence of a general molecular strategy in waterfowl that prolongs the duration of Piezo2-mediated MA current. Such mechanisms, which remain to be determined, could include modification of

Piezo2 by splicing, interaction with regulatory proteins, or membrane lipid environments (Anderson et al., 2018; Coste et al., 2015; Lewis and Grandl, 2015; Qi et al., 2015; Szczot et al., 2017; Wu et al., 2017; Zheng et al., 2019).

The neuroethological basis of tactile foraging behavior is complex; in addition to numerical expansion of mechanoreceptors in TG and modification of Piezo2 function, it likely involves molecular tuning at various levels of the peripheral nervous system and the CNS (Gutiérrez-Ibáñez et al., 2009; Wylie et al., 2015). These could involve innervation density in the bill skin, receptive field area size and sensitivity, morphological features of the bill, and central representation and processing, which remain to be explored.

STAR★METHODS

Detailed methods are provided in the online version of this paper and include the following:

- KEY RESOURCES TABLE
- CONTACT FOR REAGENT AND RESOURCE SHARING
- EXPERIMENTAL MODEL AND SUBJECT DETAILS
- METHOD DETAILS
  - Patch-clamp electrophysiology in neurons
  - Ratiometric live-cell calcium imaging
  - RNA *in situ* hybridization
  - Transcriptome analysis
- QUANTIFICATION AND STATISTICAL ANALYSIS
- DATA AND SOFTWARE AVAILABILITY

SUPPLEMENTAL INFORMATION

Supplemental Information can be found with this article online at https://doi.org/10.1016/j.celrep.2019.01.100.

ACKNOWLEDGMENTS

We thank members of the Gracheva and Bagriantsev laboratories for their contributions throughout the project, Kate McGrew and Alicia Berlin from the USGS Patuxent Wildlife Research Center, Nick Tiberio from Livingston Ripley Waterfowl Conservancy, and the Metzer Farms staff for providing bird eggs. E.R.S. is a postdoctoral fellow of the Arnold and Mabel Beckman Foundation. E.O.A. is an Edward L. Tatum fellow and was partially supported by the Gruber Foundation. V.V.F. is a James Hudson Brown-Alexander B. Coxe fellow. This study was partly funded by NIH grant 1R01NS091300-01A1 (to E.O.G.), NSF CAREER grant 1453167 (to S.N.B.), and NIH grant 1R01NS097547-01A1 (to S.N.B.).

AUTHOR CONTRIBUTIONS

E.R.S., E.O.A., V.V.F., M.M., Y.A.N., E.O.G., and S.N.B. designed and performed experiments and collected and analyzed data. E.O.G. and S.N.B. wrote the paper, with contributions from all authors. E.O.G. and S.N.B. conceived the study and provided guidance and supervision throughout the project.

DECLARATION OF INTERESTS

The authors declare no competing interests.

Received: October 31, 2018
Revised: December 20, 2018
Accepted: January 25, 2019
Published: February 19, 2019

SUPPORTING CITATIONS

The following references appear in the Supplemental Information: Buckner et al. (2018); Gonzalez et al. (2009).

REFERENCES

Anderson, E.O., Schneider, E.R., and Bagriantsev, S.N. (2017). Piezo2 in cutaneous and proprioceptive mechanotransduction in vertebrates. Curr. Top. Membr. *79*, 197–217.

Anderson, E.O., Schneider, E.R., Matson, J.D., Gracheva, E.O., and Bagriantsev, S.N. (2018). TMEM150C/Tentonin3 is a regulator of mechano-gated ion channels. Cell Rep. *23*, 701–708.

Avilova, K.V. (2017). The feeding behavior of the mallard (*Anas platyrhynchos*) in the darkness. Sens. Syst. *31*, 139–143.

Avilova, K.V. (2018). Spatial organization of the epithelial structures in the bill tip organ of waterfowl (Anseriformes, Aves). Biol. Bull. Rev. *8*, 234–244.

Avilova, K.V., Fedorenko, A.G., and Lebedeva, N.V. (2018). The mechanoreceptor organs of the lamellirostral birds (Anseriformes, Aves). Biol. Bull. *45*, 51–60.

Berkhoudt, H. (1980). The morphology and distribution of cutaneous mechanoreceptors (Herbst and Grandry corpuscles) in bill and tongue of the mallard (*Anas platyrhynchos* L.). Neth. J. Zool. *30*, 1–34.

Bolger, A.M., Lohse, M., and Usadel, B. (2014). Trimmomatic: a flexible trimmer for Illumina sequence data. Bioinformatics *30*, 2114–2120.

Buckner, J.C., Ellingson, R., Gold, D.A., Jones, T.L., and Jacobs, D.K. (2018). Mitogenomics supports an unexpected taxonomic relationship for the extinct diving duck *Chendytes lawi* and definitively places the extinct Labrador duck. Mol. Phylogenet. Evol. *122*, 102–109.

Cabo, R., Alonso, P., San José, I., Vázquez, G., Pastor, J.F., Germanà, A., Vega, J.A., and García-Suárez, O. (2015). Brain-derived neurotrofic factor and its receptor TrkB are present, but segregated, within mature cutaneous Pacinian corpuscles of *Macaca fascicularis*. Anat. Rec. (Hoboken) *298*, 624–629.

Calavia, M.G., Feito, J., López-Iglesias, L., de Carlos, F., García-Suarez, O., Pérez-Piñera, P., Cobo, J., and Vega, J.A. (2010). The lamellar cells in human Meissner corpuscles express TrkB. Neurosci. Lett. *468*, 106–109.

Catania, K.C. (2011). The sense of touch in the star-nosed mole: from mechanoreceptors to the brain. Philos. Trans. R. Soc. Lond. B Biol. Sci. *366*, 3016–3025.

Catania, K.C., and Remple, F.E. (2005). Asymptotic prey profitability drives star-nosed moles to the foraging speed limit. Nature *433*, 519–522.

Coste, B., Crest, M., and Delmas, P. (2007). Pharmacological dissection and distribution of NaN/Nav1.9, T-type Ca2+ currents, and mechanically activated cation currents in different populations of DRG neurons. J. Gen. Physiol. *129*, 57–77.

Coste, B., Mathur, J., Schmidt, M., Earley, T.J., Ranade, S., Petrus, M.J., Dubin, A.E., and Patapoutian, A. (2010). Piezo1 and Piezo2 are essential components of distinct mechanically activated cation channels. Science *330*, 55–60.

Coste, B., Murthy, S.E., Mathur, J., Schmidt, M., Mechioukhi, Y., Delmas, P., and Patapoutian, A. (2015). Piezo1 ion channel pore properties are dictated by C-terminal region. Nat. Commun. *6*, 7223.

Dhandapani, R., Arokiaraj, C.M., Taberner, F.J., Pacifico, P., Raja, S., Nocchi, L., Portulano, C., Franciosa, F., Maffei, M., Hussain, A.F., et al. (2018). Control of mechanical pain hypersensitivity in mice through ligand-targeted photoablation of TrkB-positive sensory neurons. Nat. Commun. *9*, 1640.

Dobin, A., Davis, C.A., Schlesinger, F., Drenkow, J., Zaleski, C., Jha, S., Batut, P., Chaisson, M., and Gingeras, T.R. (2013). STAR: ultrafast universal RNA-seq aligner. Bioinformatics *29*, 15–21.

Drobney, R.D., and Fredrickson, L.H. (1979). Food selection by wood ducks in relation to breeding status. J. Wildl. Manage. *43*, 109–120.

Fleming, M.S., Li, J.J., Ramos, D., Li, T., Talmage, D.A., Abe, S.I., Arber, S., and Luo, W. (2016). A RET-ER81-NRG1 signaling pathway drives the development of Pacinian corpuscles. J. Neurosci. *36*, 10337–10355.

García-Suárez, O., Montaño, J.A., Esteban, I., González-Martínez, T., Alvarez-Abad, C., López-Arranz, E., Cobo, J., and Vega, J.A. (2009). Myelin basic protein-positive nerve fibres in human Meissner corpuscles. J. Anat. *214*, 888–893.

Gerhold, K.A., Pellegrino, M., Tsunozaki, M., Morita, T., Leitch, D.B., Tsuruda, P.R., Brem, R.B., Catania, K.C., and Bautista, D.M. (2013). The star-nosed mole reveals clues to the molecular basis of mammalian touch. PLoS ONE *8*, e55001.

Gonzalez, J., Duttmann, H., and Wink, M. (2009). Phylogenetic relationships based on two mitochondrial genes and hybridization patterns in Anatidae. J. Zool. *279*, 310–318.

Gonzalez-Martinez, T., Perez-Piñera, P., Díaz-Esnal, B., and Vega, J.A. (2003). S-100 proteins in the human peripheral nervous system. Microsc. Res. Tech. *60*, 633–638.

Gottschaldt, K.-M. (1974). The physiological basis of tactile sensibility in the beak of geese. J. Comp. Physiol. *95*, 29–47.

Gracheva, E.O., and Bagriantsev, S.N. (2015). Evolutionary adaptation to thermosensation. Curr. Opin. Neurobiol. *34*, 67–73.

Gutiérrez-Ibáñez, C., Iwaniuk, A.N., and Wylie, D.R. (2009). The independent evolution of the enlargement of the principal sensory nucleus of the trigeminal nerve in three different groups of birds. Brain Behav. Evol. *74*, 280–294.

Heidenreich, M., Lechner, S.G., Vardanyan, V., Wetzel, C., Cremers, C.W., De Leenheer, E.M., Aránguez, G., Moreno-Pelayo, M.A., Jentsch, T.J., and Lewin, G.R. (2011). KCNQ4 K(+) channels tune mechanoreceptors for normal touch sensation in mouse and man. Nat. Neurosci. *15*, 138–145.

Hu, J., and Lewin, G.R. (2006). Mechanosensitive currents in the neurites of cultured mouse sensory neurones. J. Physiol. *577*, 815–828.

Kobayashi, K., Fukuoka, T., Obata, K., Yamanaka, H., Dai, Y., Tokunaga, A., and Noguchi, K. (2005). Distinct expression of TRPM8, TRPA1, and TRPV1 mRNAs in rat primary afferent neurons with adelta/c-fibers and colocalization with trk receptors. J. Comp. Neurol. *493*, 596–606.

Kurganov, E., Zhou, Y., Saito, S., and Tominaga, M. (2014). Heat and AITC activate green anole TRPA1 in a membrane-delimited manner. Pflugers Arch. *466*, 1873–1884.

Lallemend, F., and Ernfors, P. (2012). Molecular interactions underlying the specification of sensory neurons. Trends Neurosci. *35*, 373–381.

Lewis, A.H., and Grandl, J. (2015). Mechanical sensitivity of Piezo1 ion channels can be tuned by cellular membrane tension. eLife *4*, e12088.

Liao, Y., Smyth, G.K., and Shi, W. (2014). featureCounts: an efficient general purpose program for assigning sequence reads to genomic features. Bioinformatics *30*, 923–930.

Luo, W., Enomoto, H., Rice, F.L., Milbrandt, J., and Ginty, D.D. (2009). Molecular identification of rapidly adapting mechanoreceptors and their developmental dependence on ret signaling. Neuron *64*, 841–856.

McCarter, G.C., Reichling, D.B., and Levine, J.D. (1999). Mechanical transduction by rat dorsal root ganglion neurons *in vitro*. Neurosci. Lett. *273*, 179–182.

McNeil, R., Drapeau, P., and Goss-Custard, J.D. (1992). The occurrence and adaptive significance of nocturnal habits in waterfowl. Biol. Rev. Camb. Philos. Soc. *67*, 381–419.

Minett, M.S., Nassar, M.A., Clark, A.K., Passmore, G., Dickenson, A.H., Wang, F., Malcangio, M., and Wood, J.N. (2012). Distinct Nav1.7-dependent pain sensations require different sets of sensory and sympathetic neurons. Nat. Commun. *3*, 791.

Murthy, S.E., Loud, M.C., Daou, I., Marshall, K.L., Schwaller, F., Kühnemund, J., Francisco, A.G., Keenan, W.T., Dubin, A.E., Lewin, G.R., and Patapoutian, A. (2018). The mechanosensitive ion channel Piezo2 mediates sensitivity to mechanical pain in mice. Sci. Transl. Med. *10*, eaat9897.

Prato, V., Taberner, F.J., Hockley, J.R.F., Callejo, G., Arcourt, A., Tazir, B., Hammer, L., Schad, P., Heppenstall, P.A., Smith, E.S., and Lechner, S.G. (2017). Functional and molecular characterization of mechanoinsensitive "silent" nociceptors. Cell Rep. *21*, 3102–3115.

Qi, Y., Andolfi, L., Frattini, F., Mayer, F., Lazzarino, M., and Hu, J. (2015). Membrane stiffening by STOML3 facilitates mechanosensation in sensory neurons. Nat. Commun. *6*, 8512.

Ranade, S.S., Woo, S.H., Dubin, A.E., Moshourab, R.A., Wetzel, C., Petrus, M., Mathur, J., Bégay, V., Coste, B., Mainquist, J., et al. (2014). Piezo2 is the major transducer of mechanical forces for touch sensation in mice. Nature *516*, 121–125.

Ranade, S.S., Syeda, R., and Patapoutian, A. (2015). Mechanically activated ion channels. Neuron *87*, 1162–1179.

Robinson, M.D., McCarthy, D.J., and Smyth, G.K. (2010). edgeR: a Bioconductor package for differential expression analysis of digital gene expression data. Bioinformatics *26*, 139–140.

Rugiero, F., Drew, L.J., and Wood, J.N. (2010). Kinetic properties of mechanically activated currents in spinal sensory neurons. J. Physiol. *588*, 301–314.

Saito, S., Banzawa, N., Fukuta, N., Saito, C.T., Takahashi, K., Imagawa, T., Ohta, T., and Tominaga, M. (2014). Heat and noxious chemical sensor, chicken TRPA1, as a target of bird repellents and identification of its structural determinants by multispecies functional comparison. Mol. Biol. Evol. *31*, 708–722.

Saxod, R. (1978). Development of cutaneous sensory receptors in birds. In Development of Sensory System, C.M. Bate, ed. (Springer-Verlag), pp. 337–417.

Saxod, R. (1996). Ontogeny of the cutaneous sensory organs. Microsc. Res. Tech. *34*, 313–333.

Schneider, E.R., Mastrotto, M., Laursen, W.J., Schulz, V.P., Goodman, J.B., Funk, O.H., Gallagher, P.G., Gracheva, E.O., and Bagriantsev, S.N. (2014). Neuronal mechanism for acute mechanosensitivity in tactile-foraging waterfowl. Proc. Natl. Acad. Sci. USA *111*, 14941–14946.

Schneider, E.R., Gracheva, E.O., and Bagriantsev, S.N. (2016). Evolutionary specialization of tactile perception in vertebrates. Physiology (Bethesda) *31*, 193–200.

Schneider, E.R., Anderson, E.O., Mastrotto, M., Matson, J.D., Schulz, V.P., Gallagher, P.G., LaMotte, R.H., Gracheva, E.O., and Bagriantsev, S.N. (2017). Molecular basis of tactile specialization in the duck bill. Proc. Natl. Acad. Sci. USA *114*, 13036–13041.

Szczot, M., Pogorzala, L.A., Solinski, H.J., Young, L., Yee, P., Le Pichon, C.E., Chesler, A.T., and Hoon, M.A. (2017). Cell-type-specific splicing of Piezo2 regulates mechanotransduction. Cell Rep. *21*, 2760–2771.

Szczot, M., Liljencrantz, J., Ghitani, N., Barik, A., Lam, R., Thompson, J.H., Bharucha-Goebel, D., Saade, D., Necaise, A., Donkervoort, S., et al. (2018). PIEZO2 mediates injury-induced tactile pain in mice and humans. Sci. Transl. Med. *10*, eaat9892.

Tanaka, B.S., Nguyen, P.T., Zhou, E.Y., Yang, Y., Yarov-Yarovoy, V., Dib-Hajj, S.D., and Waxman, S.G. (2017). Gain-of-function mutation of a voltage-gated sodium channel $Na_V1.7$ associated with peripheral pain and impaired limb development. J. Biol. Chem. *292*, 9262–9272.

Tome, M.W., and Wrubleski, D.A. (1988). Underwater foraging behavior of canvasbacks, lesse scaups, and ruddy ducks. Condor *90*, 168–172.

Wende, H., Lechner, S.G., Cheret, C., Bourane, S., Kolanczyk, M.E., Pattyn, A., Reuter, K., Munier, F.L., Carroll, P., Lewin, G.R., and Birchmeier, C. (2012). The transcription factor c-Maf controls touch receptor development and function. Science *335*, 1373–1376.

Wetzel, C., Hu, J., Riethmacher, D., Benckendorff, A., Harder, L., Eilers, A., Moshourab, R., Kozlenkov, A., Labuz, D., Caspani, O., et al. (2007). A stomatin-domain protein essential for touch sensation in the mouse. Nature *445*, 206–209.

Wu, J., Young, M., Lewis, A.H., Martfeld, A.N., Kalmeta, B., and Grandl, J. (2017). Inactivation of mechanically activated piezo1 ion channels is determined by the C-terminal extracellular domain and the inner pore helix. Cell Rep. *21*, 2357–2366.

Wylie, D.R., Gutiérrez-Ibáñez, C., and Iwaniuk, A.N. (2015). Integrating brain, behavior, and phylogeny to understand the evolution of sensory systems in birds. Front. Neurosci. *9*, 281.

Yang, L., Dong, F., Yang, Q., Yang, P.F., Wu, R., Wu, Q.F., Wu, D., Li, C.L., Zhong, Y.Q., Lu, Y.J., et al. (2017). FGF13 selectively regulates heat nociception by interacting with Nav1.7. Neuron *93*, 806–821.

Zheng, W., Gracheva, E.O., and Bagriantsev, S.N. (2019). A hydrophobic gate in the inner pore helix is the major determinant of inactivation in mechanosensitive Piezo channels. eLife *8*, e44003.

Zweers, G.A. (1977). Mechanics of the Feeding of the Mallard (*Anas platyrhynchos*, L.; Aves, Anseriformes), First Edition (S. Karger Publishers).

STAR★METHODS

KEY RESOURCES TABLE

REAGENT or RESOURCE	SOURCE	IDENTIFIER
Critical Commercial Assays		
KAPA mRNA Hyper Prep kit	Roche Sequencing	Cat# KK8581
Deposited Data		
Duck genome: *Anas platyrhynchos*	NCBI	GCF_000355885.1
Chicken genome: *Gallus gallus*	NCBI	GCF_000002315.5
Transcriptome of bird trigeminal ganglia	This paper	GEO: GSE125754
Experimental Models: Organisms/Strains		
American black duck (*Anas rubripes*)<br>Harlequin duck (*Histrionicus histrionicus*)	USGS Patuxent Wildlife Research Center	N/A
Lesser Scaup (*Aythya affinis*)		
Hooded merganser (*Lophodytes cucullatus*)	Livingston Ripley Waterfowl Conservancy	N/A
Ruddy duck (*Oxyura jamaicensis*)		
Wood duck (*Aix sponsa*)	USGS Patuxent Wildlife Research Center or Livingston Ripley Waterfowl Conservancy	N/A
Pekin duck (*Anas platyrhynchos domesticus*)	Metzer Farms	N/A
Domestic chicken (*Gallus domesticus*)		
Oligonucleotides		
Piezo2 *in situ* RNA probe primer Fwd GACAGTATCTCCAGCTGCTAC	(Schneider et al., 2014)	N/A
Piezo2 *in situ* RNA probe primer Rev TTATGGACCATCAGCCCTCCCA	(Schneider et al., 2014)	N/A
Software and Algorithms		
GraphPad Prism 7	GraphPad	RRID:SCR_002798
ImageJ	NIH	RRID:SCR_003070
pClamp	Molecular Devices	RRID:SCR_011323
MetaFluor	Molecular Devices	RRID:SCR_014294
Igor Pro 6.3	Wavemetrics	RRID:SCR_000325
TaroTools	Dr. Taro Ishikawa, Jikei University	N/A
Trimmomatic	(Bolger et al., 2014)	RRID:SCR_011848
STAR	(Dobin et al., 2013)	RRID:SCR_015899
featureCounts	(Liao et al., 2014)	RRID:SCR_012919
R	N/A	RRID:SCR_001905
edgeR (package for R)	(Robinson et al., 2010)	RRID:SCR_012802
Hmisc (package for R)	N/A	N/A

CONTACT FOR REAGENT AND RESOURCE SHARING

Further information and requests for resources and reagents should be directed to and will be fulfilled by the Lead Contact, Sviatoslav Bagriantsev (slav.bagriantsev@yale.edu).

EXPERIMENTAL MODEL AND SUBJECT DETAILS

All procedures with bird embryos were performed in compliance with the Office of Animal Research Support of Yale University (protocol 2018-11526). Fertilized Pekin duck and domestic chicken eggs were purchased from Metzer Farms, all other duck eggs were

purchased from USGS Patuxent Wildlife Research Center (Laurel, MD) or Livingston Ripley Waterfowl Conservancy (Litchfield, CT). Eggs were incubated at 37°C and 55%–75% humidity. Embryos were extracted for dissection when they had broken through the inner shell membrane (24-48 hr before hatching), corresponding to the embryonic day 25-26 (Pekin), 21-22 (Black), 28-29 (Harlequin), 25-26 (Lesser Scaup), 28-31 (Wood), 32 (Merganser), 30-31 (Ruddy), 19-21 (Chicken).

METHOD DETAILS

Patch-clamp electrophysiology in neurons

Electrophysiological experiments were performed as described earlier (Schneider et al., 2017). Embryos were decapitated, and embryonic TG were dissected in ice-cold phosphate-buffered saline, chopped with scissors in Hanks' Balanced Salt Solution (HBSS, Lonza, #10-527F), dissociated in Collagenase P (1 mg/ml in HBSS, Roche, #11213857001) for 15 minutes at 37°C, incubated in 0.25% Trypsin-EDTA (GIBCO, #25200056) at 37°C for 10 minutes and quenched in warm (37°C) DMEM+ media (standard DMEM media supplemented with 1% penicillin/streptomycin, 10% fetal bovine serum, 2mM glutamine, 4.5g/L D-glucose). Cells were gently triturated by pipetting, centrifuged 5 min at 100 x g and resuspended in DMEM+. 15μL of cell suspension was plated on coverslips coated with Matrigel (1:100 in PBS) in a 12-well cell culture plate and incubated at 37°C and 5% CO_2 for 30-45 minutes before adding 0.5 mL DMEM+ to each well. Electrophysiological recordings were conducted 1-48 hours following addition of DMEM+ by two operators (E.R.S. and E.O.A.).

Voltage-clamp recordings were acquired using pClamp software sampled at 20 kHz and low-pass filtered at 2-10 kHz using patch pipettes of 1.5 mm outer diameter borosilicate glass pulled to a tip resistance of 1.5-5 MΩ. Internal solution consisted of (in mM) 130 K-methanesulfonate, 20 KCl, 1 $MgCl_2$, 10 HEPES, 3 Na_2ATP, 0.06 Na_3GTP, 0.2 EGTA, pH 7.3, with KOH (final [K^+] = 150.5 mM). External solution contained the following (in mM): 140 NaCl, 5 KCl, 10 HEPES, 2.5 $CaCl_2$, 1 $MgCl_2$, 10 glucose (pH 7.4 with NaOH). Mechanical stimulation was performed using a blunt glass probe (2-4 μm at the tip) mounted on a pre-loaded piezo-actuator stack (Physik Instrumente Gmbh, DE), with the angle of the mechanical stimulation probe set to 32°-55° from the horizontal plane. The probe was then moved toward the cell in 1μm increments at a velocity of 800 μm/s, held in position for 150 ms, then retracted at the same velocity. Membrane potential was clamped at −60 mV. The liquid junction potential was 14.6 mV and subtracted offline. Immediately after establishing whole-cell recording resting potential was measured in I = 0 mode.

Ratiometric live-cell calcium imaging

Embryos were decapitated, and embryonic TG (E25-26) were placed in ice-cold HBSS (Lonza, #10-527F) solution, dissociated by scissors and mixed with Collagenase P (1 mg/ml in HBSS, Roche, #11213857001) for 15 min at 37°C. Collagenase was removed by aspiration and 0.25% Trypsin-EDTA (GIBCO, #25200056) was added to the cells for 10 min at 37°C. Following the removal of trypsin, neurons were mechanically dissociated by pipetting in DMEM supplemented with 10% fetal bovine serum (FBS), collected by centrifugation at 100 × g for 3 min, resuspended in DMEM with 10% FBS, plated onto the Poly-D-Lysine/Laminin covered coverslips (Corning, # 354087) and maintained at 37°C for 1–2 hr. Neurons were loaded with 10 μM Fura 2-AM (Thermo Fisher, # F1201) and 0.02% Pluronic F-127 in Ringer solution (in mM: 140 NaCl, 5 KCl, 10 HEPES, 2 $CaCl_2$, 2 $MgCl_2$, and 10 D-glucose, pH 7.4) for 30 min at room temperature and washed 3 times with Ringer solution. Live-cell ratiometric calcium imaging was performed at room temperature using Axio-Observer Z1 inverted microscope (Zeiss) equipped with an Orca-Flash4.0 camera (Hamamatsu) using Meta-Fluor software (Molecular Devices). Cells were exposed to 100 μM AITC (Sigma) mixed in Ringer's solution at constant perfusion at 5 ml/min. At the end of recordings, cells were exposed to a high-K^+ solution (in mM: 10 NaCl, 135 KCl, 10 HEPES, 2 $CaCl_2$, 2 $MgCl_2$ and 10 D-glucose) to differentiate neurons from other types of cells.

RNA *in situ* hybridization

Late-stage embryonic duck trigeminal ganglia were fixed in paraformaldehyde, sectioned at 12-15 μm, probed with digoxigenin-labeled cRNA probe against duck Piezo2 generated by T7/T3 *in vitro* transcription reactions using a 3.1-kb fragment of Pekin duck Piezo2 cDNA (primers: forward 5′-3′: GACAGTATCTCCAGCTGCTAC; 5′-3′ reverse: TTATGGACCATCAGCCCTCCCA). Signal was developed with alkaline phosphatase-conjugated anti-digoxigenin Fab fragments according to the manufacturer's instructions (Roche). Quantification was performed blind with regard to species identity.

Transcriptome analysis

Total RNA was isolated from trigeminal ganglia of bird species using the TRIzol reagent (ThermoFisher, Waltham, MA) according to manufacturer's instructions. RNA samples had RNA integrity numbers (RINs) in the range of 7.7-8.6, and Fragment Analyzer RNA Quality Numbers in the range of 7.6-9.2. Library preparation and sequencing were carried out at the Yale Center for Genome Analysis. mRNA was purified from ~500 ng total RNA with oligo-dT beads. Strand-specific sequencing libraries were prepared using the KAPA mRNA Hyper Prep kit (Roche Sequencing, Pleasanton, CA). Libraries were sequenced on Illumina HiSeq 2500 sequencer in the 75 bp paired-end sequencing mode according to manufacturer's protocols with 4 samples pooled per lane. A total of ~36-81 million sequencing read pairs per sample were obtained.

Sequencing data was processed on the Yale Center for Research Computing cluster. Raw sequencing reads were filtered and trimmed to retain high-quality reads using Trimmomatic v0.36 (Bolger et al., 2014) with default parameters. Filtered high-quality reads

from all samples were aligned to both duck and chicken reference genomes using STAR aligner v2.5.4b with default parameters (Dobin et al., 2013). Reference genomes and gene annotations were obtained from the National Center for Biotechnology Information. Duck genome: *Anas platyrhynchos* (assembly BGI_duck_1.0), annotation: NCBI Release 102. Chicken genome: *Gallus gallus* (assembly GRCg6a), annotation: NCBI Release 102. Only protein-coding genes were extracted from annotations and used for read counting. Aligned reads were counted by featureCounts program within the Subread package v1.6.2 with default parameters (Liao et al., 2014). Raw read counts were processed and converted to "reads per kilobase gene length per million mapped reads" (RPKM) values by EdgeR v3.22.3 (Robinson et al., 2010). To compare gene expression estimates between samples from different species, gene lists and corresponding RPKM values from duck and chicken gene annotations were merged based on the common gene symbol. RPKM values from 3 biological replicates within each species were averaged and used to build a matrix of pairwise Pearson *r* correlation coefficients as implemented in the rcorr tool from the Hmisc R package between all genes in the final gene annotation. Correlation coefficients between selected gene pairs were extracted from the correlation matrix.

QUANTIFICATION AND STATISTICAL ANALYSIS

Electrophysiological data from trigeminal neurons were obtained from at least two independent experiments by two experimenters. Data were collected in pClamp and analyzed in Igor Pro 6.3 (following conversion from pClamp using TaroTools) and GraphPad Prism 7.0. Data were collected from 2-6 birds for each species. The number of neurons for each species is indicated in figure legends. Inactivation kinetics of mechano-evoked currents were obtained as previously described (Schneider et al., 2017). The decaying component of the mechano-current was fit to the single-exponential decay equation: $I = \Delta I * exp\hat{}(-t/\tau_{inact})$, where ΔI is the difference between peak MA current and baseline, t is the time from the peak current (the start of the fit), and τ_{inact} is the decay constant. Summary τ_{inact} from figures represent averages from traces with the top 75% of mechano-current amplitude, as quantified previously (Coste et al., 2010). A χ^2 test was used to compare ratios of mechanosensitive neurons between species. Quantification of RNA *in situ* hybridization images was performed in ImageJ from 1610-3876 neurons from 7-17 random TG sections. Pearson *r* correlation coefficients and correlation P values were calculated using GraphPad Prism 7.0 or the rcorr tool from the Hmisc package. Transcriptomics data were obtained by sequencing trigeminal ganglia from three birds for each species.

DATA AND SOFTWARE AVAILABILITY

The accession number for the sequencing data reported in this paper is GEO: GSE125754.

Cell Reports, Volume *26*

Supplemental Information

A Cross-Species Analysis Reveals a General Role for Piezo2 in Mechanosensory Specialization of Trigeminal Ganglia from Tactile Specialist Birds

Eve R. Schneider, Evan O. Anderson, Viktor V. Feketa, Marco Mastrotto, Yury A. Nikolaev, Elena O. Gracheva, and Sviatoslav N. Bagriantsev

Supplemental Data

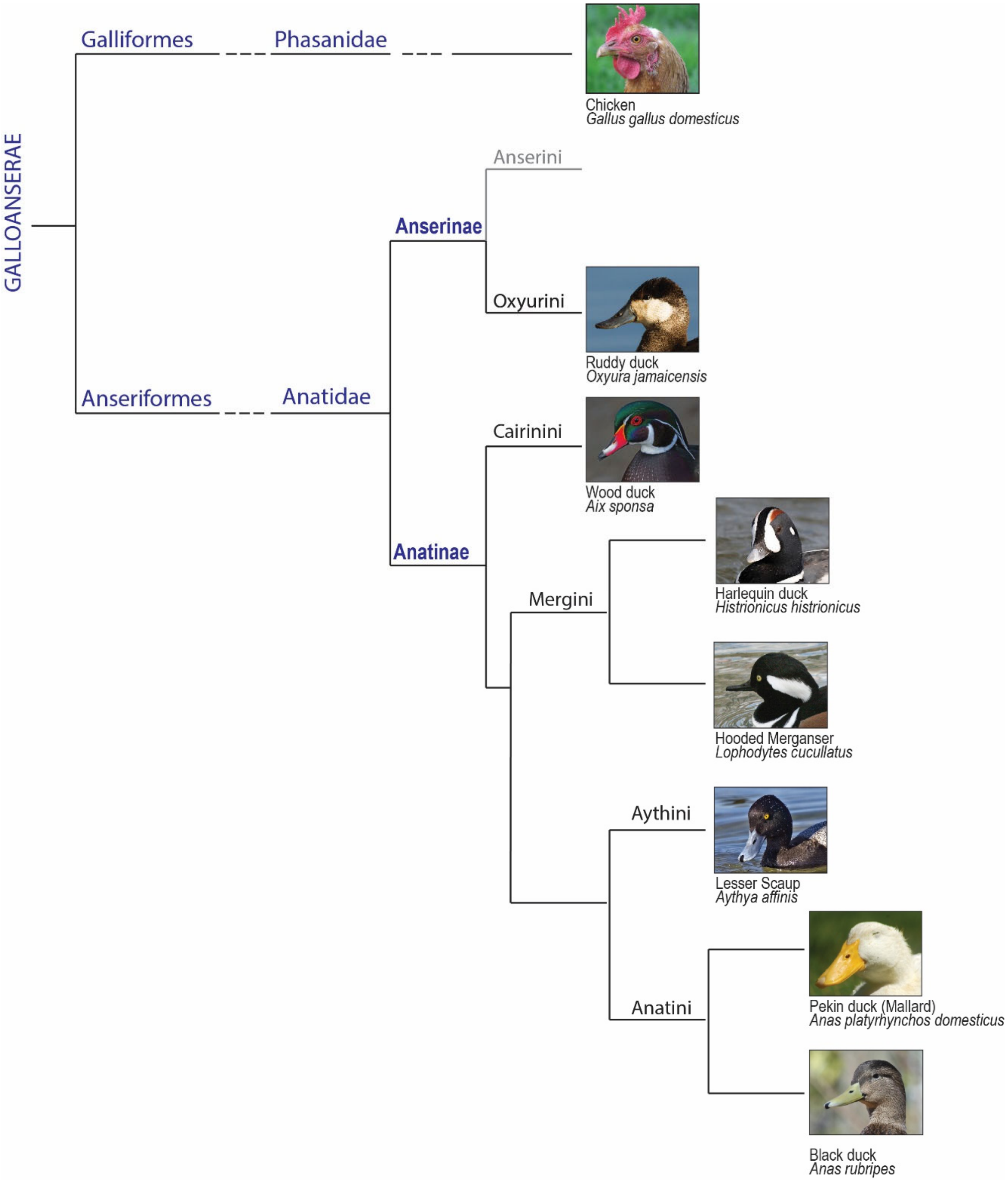

Figure S1. Phylogeny of bird species used in this study. Related to Figure 1. A simplified phylogenetic tree showing evolutionary relationship between the birds used in this study, without time representation. Anatids are representative of 2 subfamilies, 5 tribes and 6 genera. Tribes for which there are single species are not shown. The tree is adapted from (Buckner et al., 2018; Gonzalez et al., 2009). Photos courtesy of: Judy Gallagher (Wood, image cropped, CC BY 2.0); Frank Schulenburg (Ruddy, image cropped, CC BY-SA 3.0); Peter Massas (Harlequin, image cropped. CC BY-SA 2.0); Dick Daniels (Hooded Merganser and Black, image cropped, CC-BY-SA-3.0); Alan D. Wilson (Lesser Scaup, image cropped, CC-BY-SA-2.5); Eve Schneider (Pekin), Bagriantsev lab; Filip Maljković (Chicken, public domain).

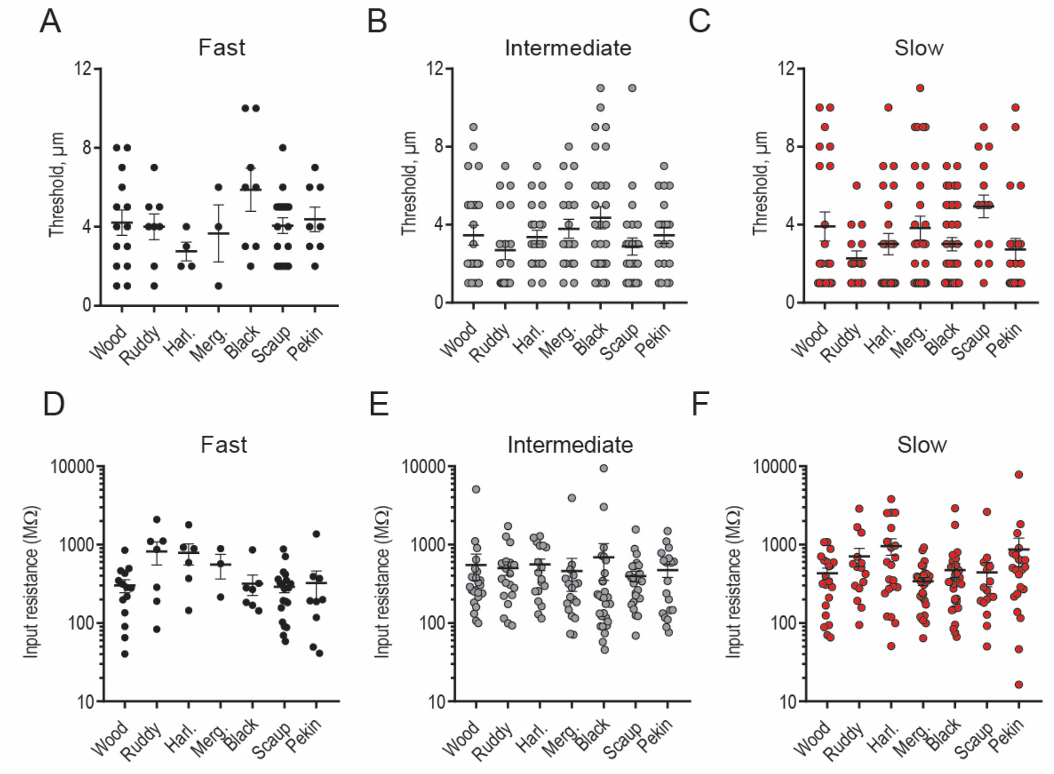

Figure S2. Threshold of mechano-current activation and input resistance of duck TG neurons. Related to Figure 1. (A–C) Quantification of the apparent MA current activation threshold from dissociated duck TG neurons. Whole-cell MA currents were elicited in response to mechanical indentation with a glass probe at E_{hold} = -74.6 mV. The threshold was determined as the first indentation to elicit a peak current greater than background noise, typically at least 50 pA above averaged baseline. Data shown as mean ± SEM. Statistical analysis (one-way ANOVA): P=0.317 (A), P=0.205 (B), P=0.063 (C). Input resistance of trigeminal neurons with the indicated types of MA current. Data shown as mean ± SEM, collected from 2-6 birds for each species. Statistical analysis (Kruskal-Wallis test): P=0.102 (D), P=0.150 (E), P=0.203 (F).

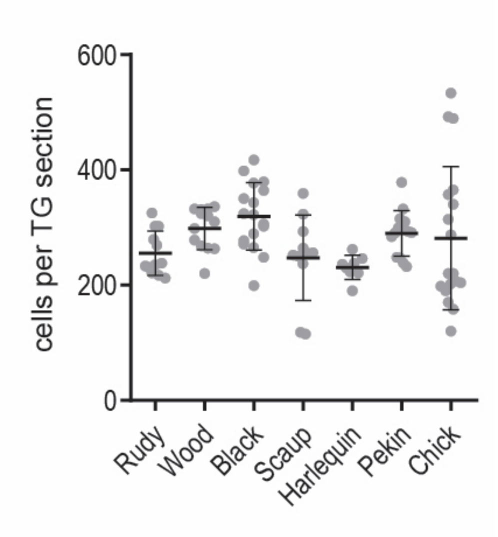

Figure S3. **Trigeminal ganglia from ducks and chicken have a similar total number of neurons. Related to Figure 2.** Quantification of the total number of cells per random TG section from indicated species. Data shown as mean ± SD, collected from ≥2 birds for each species. P≥0.05 for all sample pairs (Tukey multiple comparisons test).

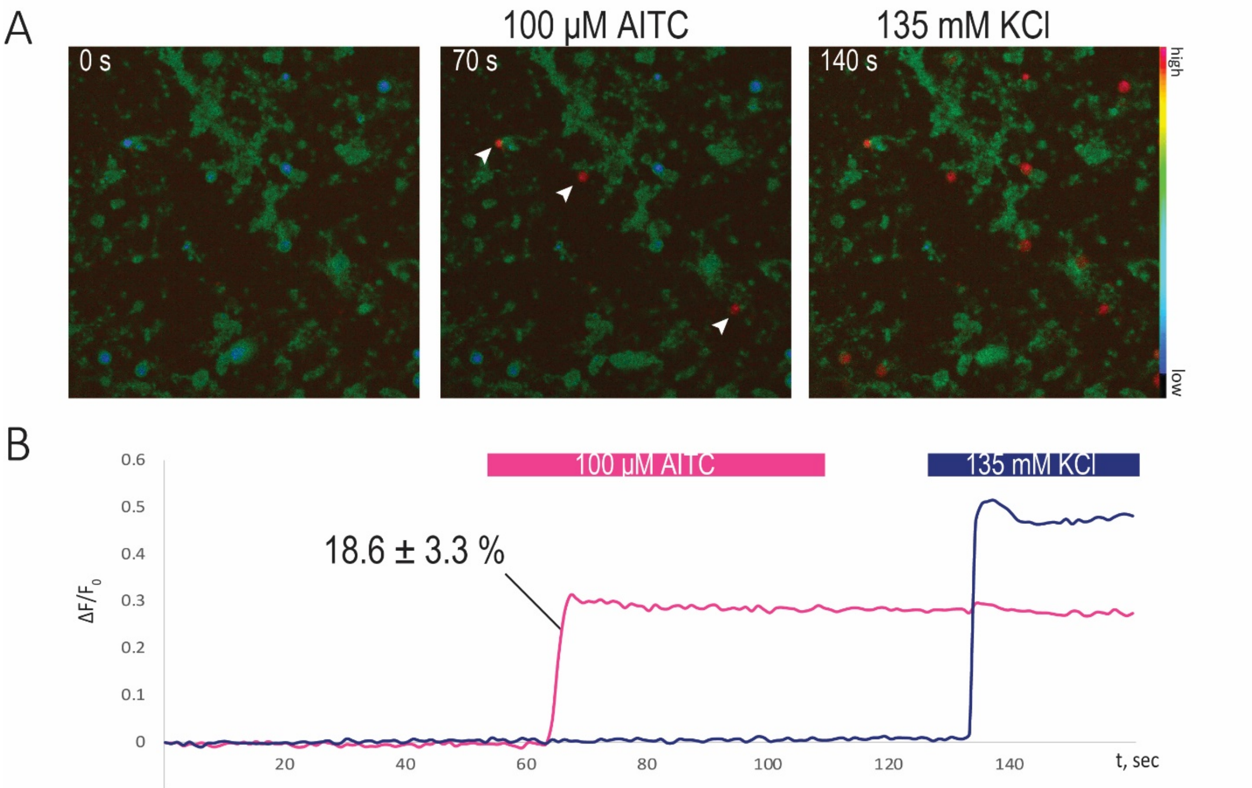

Figure S4. **Quantification of the abundance of polymodal nociceptors in Pekin duck TG. Related to Figure 4.** (A) Representative fields of view of Fura-2AM live-cell ratiometric calcium imaging of dissociated duck TG neurons in response to the application of 100 µM AITC. Arrowheads denote AITC-sensitive cells. 135 mM KCl is used to visualize all neurons in the field of view. Color coding denotes lowest and highest ratios from bottom to top. (B) Exemplar traces of calcium responses in an AITC-sensitive and insensitive cell, from the images of duck TG neurons above (mean ± SEM, n = 158 neurons from 5 coverslips, from 4 animals).
